# Theme identifiability indices in Spanish for a set of 70 *ad hoc* categorical lists

**DOI:** 10.3389/fpsyg.2024.1474494

**Published:** 2024-11-28

**Authors:** Verónica Benítez, María Angeles Alonso

**Affiliations:** ^1^Instituto Universitario de Neurociencia (IUNE), University of La Laguna, San Cristóbal de La Laguna, Spain; ^2^Facultad de Psicología y Logopedia, University of La Laguna, San Cristóbal de La Laguna, Spain; ^3^Instituto de Integración en la Comunidad (INICO), University of Salamanca, Salamanca, Spain

**Keywords:** theme identifiability, *ad hoc* categorical relationships, false memories, DRM paradigm, error editing processes

## Introduction

The field of false memories has been widely studied in cognitive psychology through the DRM paradigm (Deese, 1959; Roediger and McDermott, 1995), an experimental task used to induce false memories from materials that are conceptually and semantically related. This paradigm involves presenting lists of words that are semantically related to a non-presented critical word, with a subsequent memory test showing high levels of false recall and false recognition of that non-studied critical word. For example, after studying a list containing words, such as “butter,” “food,” and “sandwich,” it is likely that in a subsequent free recall or recognition test, the word “bread” will be mistakenly identified as studied.

False memory is generated, in part, by the relationship between the list words and the critical word (Gallo, 2010; Roediger and Gallo, 2016). This phenomenon has been explained by two theories: the fuzzy-trace theory (FTT) (Brainerd and Reyna, 1998), and the activation-monitoring framework (AMF) (Roediger et al., 2001). Both theories agree that, understanding the production of false memories requires considering two complementary processes: an error inflation process, identified as gist encoding in FTT and as activation in AMF; and an error editing process, identified as monitoring in AMF and as recollection rejection in FTT (Brainerd and Reyna, 2002).

According to FTT (Brainerd and Reyna, 1998), “gist” refers to the general theme extracted from studied material. When a list of words related to a non-presented critical word is studied, both literal and semantic information are encoded. In a subsequent memory test, these literal and semantic memory traces operate simultaneously, providing information about the items. The retrieval of semantic information may lead to considering the critical word as having been studied due to its similarity to the presented words. However, the retrieval of literal information about the studied associates can counteract this effect by providing evidence that the critical word was not studied, a process known as recollection rejection (Brainerd and Reyna, 2002).

According to the AMF (Roediger et al., 2001), two processes work together to produce false memory: activation and monitoring. Studying a list of words can trigger activation that spreads through the lexical-semantic system, creating implicit associations between interconnected words. This activation is moderated by a subsequent monitoring process that helps distinguishing between correct recall (studied words) and false memories (non-studied words).

Even when considering different perspectives, both FTT and AMF agree that presenting a list of associates words activates a non-presented critical word, leading to error inflation. If the error inflation process is not accompanied by its corresponding error editing process, or if this process fails, false recall or false recognition may occur (Arndt and Gould, 2006). Therefore, studying the strategies used to avoid false memories is crucial for understanding the mechanisms underlying their formation.

Theme identifiability of a list is one of the essential factors involved in this error editing process (Carneiro et al., 2009). In their normative study in Portuguese, they provided theme identifiability norms for 40 DRM associative lists selected from Albuquerque's (2005) study. Participants were presented with the lists and asked to generate a word that best described the general theme of each list. To study the effect of this factor on the production of false memories, they selected those lists with the highest and lowest levels of theme identifiability. The results showed that lists with high identifiability of the critical word as the theme produced lower levels of false recall and false recognition compared to lists where the critical word was not as easily identifiable. According to Carneiro et al. (2012), this outcome was attributed to an error editing strategy called “Identify to reject,” which involves several stages: detecting that all the words in a list are related to a common theme, identifying the word that best describes the theme but is not present in the list, keeping it in mind to avoid recalling it in the future, and consequently, reducing false memories.

This pattern has been observed in other studies using lists with an associative structure (Beato et al., 2023; Carneiro and Fernandez, 2013; Carneiro et al., 2012). Additionally, there are other normative studies that provide theme identifiability indices for associative lists in Spanish (Beato and Cadavid, 2016) and in English (Neuschatz et al., 2003).

Most studies on false memories and error editing mechanisms using the DRM paradigm employ lists with an associative structure. However, *ad hoc* categorical relationships are less explored in the literature. *Ad hoc* categories are spontaneously constructed to achieve a specific goal in a given context, and their elements can come from different taxonomic categories (e.g., “*Things that can fall on your head*”) (Barsalou, 1983). Both common and *ad hoc* categories can lead to similar memory distortions. While false memories are typically more pronounced for common categories, they are still robust for *ad hoc* categories (Soro et al., 2017).

The mechanisms for avoiding memory distortions in such lists are not well understood. *Ad hoc* categories provide a valuable tool for studying situated concept representations, which are characterized by their flexibility and dynamism (Barsalou, 2005). Their use allows researchers to explore how individuals organize and retrieve information when categories are not predefined but emerge from context. This is particularly relevant for understanding how memory adapts to new information and situations, adding depth to theoretical debates on flexible concept representation.

In the study with associative lists by Carneiro et al. (2009), the percentages of identification for critical words ranged from 1 to 77%. However, Soro et al. (2017) indicate that, unlike associative lists, in *ad hoc* categorical structured lists, theme identification typically refers to identifying the category label rather than the critical word itself. They used two criteria: exact identifiability, where participants identified the original theme of the lists (e.g., “Materials that cover the ground” for the category “Things that can be walked upon”), and comprehensive identifiability, where participants identified a theme that could include the critical word (e.g., a label that includes the critical word “grass” for “Things that can be walked upon”). Both criteria are important for describing our findings.

To our knowledge, no previous studies have addressed false memories or theme identification with *ad hoc* categories in Spanish. Therefore, the aim of this research was to obtain theme identifiability indices for 70 lists that maintained *ad hoc* categorical relationships with a non-presented critical word using the DRM paradigm. These lists were created based on a normative study of *ad hoc* categories conducted in Spanish, which, to our knowledge, is the first of its kind in this language. Additionally, this research aims to lay the groundwork for studying the underlying mechanisms of error editing processes in lists with *ad hoc* categorical relationships in Spanish.

In future research, these data may help in understanding the role of theme identifiability in the formation of false memories. Moreover, having these indices will enable more accurate predictions and better control over the experimental materials.

## Method

### Participants

A total of 188 students from the Psychology degree program at the University of La Laguna participated. All participants were native Spanish speakers (146 women, 42 men; mean age = 20.14, SD = 2.94).

### Materials

The material consisted of 70 *ad hoc* categorical lists, each containing 10 words. Both the critical words and their corresponding associates were extracted from a normative study conducted to collect data on *ad hoc* categories in Spanish (Alonso et al., in preparation; Benítez et al., 2022). *Ad hoc* categories were selected from various normative studies in English and Portuguese and then translated into Spanish (Barsalou, 1982, 1983, 1985; Hough and Pierce, 1989; Soro and Ferreira, 2017; Vallée-Tourangeau et al., 1998; Van Overschelde et al., 2004). The general procedure used in the Spanish normative study was similar to that used by Battig and Montague (1969), with the exception that in the present study both the presentation of the material and the collection for responses were done by computer (see Van Overschelde et al., 2004). The participants were instructed to generate as many exemplars as possible for each category within 1 min. This study, currently in preparation, will provide indices of frequency, rank, and lexical availability for the exemplars of each category.

The lists were constructed based on the frequencies obtained from the normative study. Critical words were selected as those with the highest frequency within each category, while their associates were the next most frequent words. Care was taken to ensure that the critical words did not appear in more than one list and that no associate was repeated across lists. The selected critical words were primarily nouns (with only four being verbs and one adjective), ranging from 1 to 5 syllables, with a mean frequency of occurrence in Spanish of 60.86 per million (Alonso et al., 2011).

The 70 lists were divided into 5 blocks: 4 blocks containing 15 lists each and one block with 10 lists. For the theme identification test, a booklet was prepared with several pages. The first page collected participant information (name, age, gender, and degree). The second page included practice examples to familiarize participants with the task. The remaining pages were dedicated to the experimental lists. Each page displayed the list number and had three blank spaces for participants to write the word or words they believed identified the theme of the list (up to three), along with a Likert scale to indicate their confidence in whether the word given in the first position represented the list's theme.

### Procedure

Data collection took place in November 2023 during group sessions, each consisting of approximately 35 participants and lasting around 30 min. Each group studied 15 lists, except for one group that studied only 10 lists. The order of list presentation within each group was randomly determined.

Participants began by completing the demographic information on the first page of the booklet and then received instructions similar to those used in previous studies (Carneiro et al., 2009; Neuschatz et al., 2003). They were shown a series of lists in a PowerPoint presentation, with one word displayed every 2 s. Before the start of the experimental session, and to ensure participants fully understood the task, two practice trials were conducted. These trials involved the same task as the main session: following the presentation of each list, participants had 50 s to generate up to three words that they believed best described the theme of the list. They also provided a confidence judgment on how certain they were that the first word given represented the list's theme, using a Likert scale ranging from 1 (“not very confident”) to 5 (“very confident”).

Before each list, a message appeared on the screen indicating the list number to be presented. The process of presenting the list and identifying the theme was repeated until the experimental session was complete.

## General description of the database

The spreadsheet file accompanying this report (Theme_Identifiability_Ad_hoc_Spanish.xls) consists of five sheets. The first sheet, “Theme identifiability,” contains the raw data for each participant. It includes the 70 critical words and their respective lists of 10 *ad hoc* associates. The first column shows the participant number, the second column identifies the list, the third column specifies the type of relationship of the lists (*ad hoc*), and the fourth column indicates the type of word (studied vs. critical). The fifth column contains the words themselves, while the sixth column provides the English translations of the critical and studied words. Adjacent columns include all responses provided by participants in the first, second, and third positions, as well as the confidence judgements related to the first response. Additionally, intrusions are noted—i.e., words from a study list that a participant mistakenly identified as the theme of that list, and therefore are not considered valid responses.

The second sheet, “First word,” summarizes the total count of responses given in the first position for each list. It includes all the words generated by participants as the theme in the first position, associated with their respective critical word and list number. Additionally, it provides the English translation of the critical word, the total number of participants who responded to each list, the number and percentage of participants who identified a word as the theme, and the mean confidence judgement for each first-word response. Intrusions are also noted, including quantity and incorrectly identified words.

The third and fourth sheets, “Second word” and “Third word,” respectively, are dedicated to responses given in the second and third positions. The layout is identical to the previous sheets, but it does not include the column for mean confidence judgement. The fifth sheet, “Summary,” presents the final summary, showing the most frequently identified theme for the first, second, and third positions for each list.

## Descriptive analysis

Responses recorded in the database were maintained in their original format, with corrections made only for spelling errors. Singular/plural and masculine/feminine forms of words were counted separately.

Supplementary Table 1 shows the 70 critical words with their corresponding list identifier and English translation, the number of participants who responded to each list, the number of different themes given as the first response, the percentage of participants who indicated the critical word (comprehensive identification) as the theme of the list in the first position, and the mean confidence rating for the critical word as the first response. Additionally, it provides the percentages of participants who identified the *ad hoc* category label (exact identification) in the first position, as well as the mean confidence rating for these first-position responses.

Theme identifiability is a crucial factor in the error editing process and thus influences the formation of false memories (Carneiro et al., 2009; Soro et al., 2017). However, the study of false memories in *ad hoc* categorical lists and the factors contributing to their formation remain unexplored in Spanish. Therefore, this study aimed to provide theme identifiability indices for 70 *ad hoc* lists within the framework of the DRM paradigm in Spanish.

When examining identifiability levels using the same approach as in studies with associative lists (Carneiro et al., 2009), where the critical word is considered as the theme, the levels of identifiability are relatively low. Regarding comprehensive identifiability, the critical word was identified as a theme in the first position in only 10 out of the 70 lists, with identification percentages ranging from 2.1 to 17.2%. On average, the critical word was identified as the theme 0.98% (SD = 3.11) of the time in the first position across all 70 lists. When considering only where the critical word was identified, this average increased to 6.88% (SD = 5.39), with a mean confidence rating of 3.57 (SD = 1.29). Given the *ad hoc* category exemplars can come from different categories, their membership is not immediately apparent without context, making the activation of the critical word challenging.

In contrast, exact identifiability showed higher levels of identification. The theme of 59 lists (ranging from 2.1 to 87.5%) was identified in the first position, demonstrating a significant increase in identification compared to when only the critical word was considered. Participants often generated words that, while not precisely matching the category labels, were related to them, suggesting some thematic processing even if not explicitly expressed. On average, the theme was identified 22.22% (SD = 23.75) of the time in the first position across all 70 lists. When considering only the lists where the theme was identified, this average rose to 26.36% (SD = 23.67), with a mean confidence rating of 3.98 (SD = 0.70) for the first response.

According to Carneiro et al. (2009), in associative lists, the level of identification of the critical word as the theme is inversely related to the occurrence of false memories. Identifying the critical word as the theme triggers an error editing process that mitigates false memories in subsequent memory tests. However, this assumption may not hold true for categorical lists, particularly *ad hoc* categorical lists, where theme identifiability might not exert the same effect on the error editing process.

Soro et al. (2017) suggested that for false memories to occur with *ad hoc* categories, a positive relationship with theme identifiability might be necessary due to the inherent variability among exemplars. Their study found no correlation between false recognition and theme identifiability. Instead, false recognition was influenced more by individual factors such as experience or creative thinking. Some participants generated more associations between exemplars, leading to an increased likelihood of errors in a subsequent recognition test.

Our results suggest that context plays a fundamental role in theme identification within *ad hoc* categories, particularly concerning comprehensive identifiability. Therefore, considering the findings of Soro et al. (2017), the participant's ability to integrate exemplars into an appropriate context may significantly influence the activation of critical words. This aligns with the notion that memory is a dynamic process shaped by contextual factors (Barsalou, 2005).

## Conclusions

The present study provides valuable data on theme identifiability for a wide range of *ad hoc* categorical lists, underscoring the need for a different approach when studying error editing processes in this context. Our findings reveal a crucial aspect of *ad hoc* categories: while traditional associative lists tend to exhibit reduced false memories due to clearer theme identifiability, the inherent variability and contextual emergence of *ad hoc* categories introduce complexities in memory retrieval that merit further investigation. This area represents a promising opportunity for advancing our understanding of false memories.

## Data Availability

The database (Theme_Identifiability_Ad_hoc_Spanish.xls) for this study can be accessed through the following link: https://data.mendeley.com/datasets/nyb7gj59gk/1.

## References

[B1] Albuquerque P. B. (2005). Produção de evocações e reconhecimentos falsos em 100 listas de palavras associadas portuguesas. Lab. Psicol. 3, 3–12. 10.14417/lp.766

[B2] Alonso M. A. Fernandez A. Díez E. (2011). Oral frequency norms for 67,979 Spanish words. Behav. Res. Methods 43, 449–458. 10.3758/s13428-011-0062-3 21416306

[B3] Arndt J. Gould C. (2006). An examination of two-process theories of false recognition. Memory 14, 814–833. 10.1080/09658210600680749 16938694

[B4] Barsalou L. W. (1982). Context-independent and context-dependent information in concepts. Mem. Cogn. 10, 82–93. 10.3758/BF03197629 7087773

[B5] Barsalou L. W. (1983). *Ad hoc* categories. Mem. Cogn. 11, 211–227. 10.3758/BF03196968 6621337

[B6] Barsalou L. W. (1985). Ideals, central tendency, and frequency of instantiation as determinants of graded structure in categories. J. Exp. Psychol. Learn. Mem. Cogn. 11, 629–654. 10.1037//0278-7393.11.1-4.629 2932520

[B7] Barsalou L. W. (2005). “Situated conceptualization,” in Handbook of Categorization in Cognitive Science, eds. H. Cohen, and C. Lefebvre (St. Louis, MO: Elsevier), 619–650. 10.1016/B978-008044612-7/50083-4

[B8] Battig W. F. Montague W. E. (1969). Category norms for verbal items in 56 categories: a replication and extension of the Connecticut category norms. J. Exp. Psychol. 80(3, Pt.2), 1–46. 10.1037/h0027577

[B9] Beato M. S. Cadavid S. (2016). Normative study of theme identifiability: instructions with and without explanation of the false memory effect. Behav. Res. Methods 48, 1252–1265. 10.3758/s13428-015-0652-6 26424441

[B10] Beato M. S. Suarez M. Cadavid S. (2023). Disentangling the effects of backward/forward associative strength and theme identifiability in false memory. Psicothema 35, 178–188. 10.7334/psicothema2022.288 37096412

[B11] Benítez V. Alonso M. A. Fernandez A. Díez E. (2022). “Categorías Ad Hoc: Normas para 71 categorías en Español,” in [Póster] La Mente Léxica: I Jornada Internacional sobre Procesamiento Léxico-Semántico, Universidad de Salamanca, España.

[B12] Brainerd C. J. Reyna V. F. (1998). Fuzzy-trace theory and children's false memories. J. Exp. Child Psychol. 71, 81–129. 10.1006/jecp.1998.2464 9843617

[B13] Brainerd C. J. Reyna V. F. (2002). Fuzzy-trace theory and false memory. Curr. Dir. Psychol. Sci. 11, 164–169. 10.1111/1467-8721.00192

[B14] Carneiro P. Fernandez A. (2013). Retrieval dynamics in false recall: revelations from identifiability manipulations. Psychon. Bull. Rev. 20, 488–495. 10.3758/s13423-012-0361-4 23299305

[B15] Carneiro P. Fernandez A. Dias A. R. (2009). The influence of theme identifiability. Mem. Cogn. 37, 115–129. 10.3758/MC.37.2.115 19223562

[B16] Carneiro P. Fernandez A. Diez E. Garcia-Marques L. Ramos T. Ferreira M. B. (2012). “Identify-to-reject”: a specific strategy to avoid false memories in the DRM paradigm. Mem. Cogn. 40, 252–265. 10.3758/s13421-011-0152-6 21971606

[B17] Deese J. (1959). On the prediction of occurrence of particular verbal intrusions in immediate recall. J. Exp. Psychol. 58, 17–22. 10.1037/h0046671 13664879

[B18] Gallo D. A. (2010). False memories and fantastic beliefs: 15 years of the DRM illusion. Mem. Cogn. 38, 833–848. 10.3758/MC.38.7.833 20921097

[B19] Hough M. S. Pierce R. S. (1989). “Contextual influences on category concept generation in aphasia,” in Clinical Aphasiology, Vol. 18, ed. T. Prescott (Boston, MA: College-Hill Press), 507–520.

[B20] Neuschatz J. S. Benoit G. E. Payne D. G. (2003). Effective warnings in the Deese-Roediger-McDermott false-memory paradigm: the role of identifiability. J. Exp. Psychol. Learn. Mem. Cogn. 29, 35–41. 10.1037//0278-7393.29.1.35 12549581

[B21] Roediger H. L. Gallo D. A. (2016). “Associative memory illusions,” in Cognitive Illusions: A Handbook on Fallacies and Biases in Thinking, Judgment and Memory, 2nd Edn, ed. R. F. Pohl (London: Routledge), 390–405.

[B22] Roediger H. L. McDermott K. B. (1995). Creating false memories: remembering words not presented in lists. J. Exp. Psychol. Learn. Mem. Cogn. 21, 803–814. 10.1037//0278-7393.21.4.803

[B23] Roediger H. L. I. I. I. Balota D. A. Watson J. M. (2001). “Spreading activation and arousal of false memories,” in The Nature of Remembering: Essays in Honor of Robert G. Crowder, eds. H. L. Roediger III, J. S. Nairne, I. Neath, and A. M. Surprenant (Washington, DC: American Psychological Association), 95–115. 10.1037/10394-006

[B24] Soro J. C. Ferreira M. B. (2017). Normas de categorias *ad hoc* para língua portuguesa. Psicologia 31, 59–68. 10.17575/rpsicol.v31i1.1285

[B25] Soro J. C. Ferreira M. B. Semin G. R. Mata A. Carneiro P. (2017). *Ad hoc* categories and false memories: memory illusions for categories created on-the-spot. J. Exp. Psychol. Learn. Mem. Cogn. 43, 1779–1792. 10.1037/xlm0000401 28383958

[B26] Vallée-Tourangeau F. Anthony S. H. Austin N. G. (1998). Strategies for generating multiple instances of common and *ad hoc* categories. Memory 6, 555–592. 10.1080/741943085 10197163

[B27] Van Overschelde J. P. Rawson K. A. Dunlosky J. (2004). Category norms: an updated and expanded version of the norms. J. Mem. Lang. 50, 289–335. 10.1016/j.jml.2003.10.00333216006

