# Supplementary material for: Theme identifiability indices in Spanish for a set of 70 ad hoc categorical lists
Source: Front Psychol. 2024 Nov 28;15:1474494. doi: 10.3389/fpsyg.2024.1474494 (PMC11638588; doi:10.3389/fpsyg.2024.1474494)
Supplement: Supplementary file 1 [file Table_1.DOCX]

**TABLE 1.** *Theme identifiability indices for 70 ad hoc lists in Spanish.*

| Nº of List | Critical Word (CW) in Spanish | English Translation | N | Nº of themes, 1º response | % Comprehensive Identifiability (CW), 1º response | Mean  Confidence CW | % Exact Identifiability, 1º response | Mean Confidence Exact Identifiability |
| --- | --- | --- | --- | --- | --- | --- | --- | --- |
| 1 | escalera | stair | 35 | 17 | 0 | 0 | 0 | 0 |
| 2 | mesa | table | 35 | 19 | 0 | 0 | 5.7 | 3.8 |
| 3 | móvil | mobile | 35 | 28 | 0 | 0 | 2.9 ^2^ | 4 |
| 4 | agua | water | 35 | 13 | 0 | 0 | 0 | 0 |
| 5 | bandera | flag | 35 | 15 | 0 | 0 | 2.9 ^2^ | 5 |
| 6 | almohada | pillow | 35 | 19 | 0 | 0 | 0 | 0 |
| 7 | hilo | thread | 35 | 9 | 0 | 0 | 2.9 | 4 |
| 8 | martillo | hammer | 35 | 13 | 0 | 0 | 0 | 0 |
| 9 | pan | bread | 35 | 4 | 0 | 0 | 5.7 ^2^ | 3.3 |
| 10 | ruido | noise | 35 | 23 | 14.3 | 2.8 | 11.4 | 3.1 |
| 11 | juguete | toy | 35 | 21 | 17.2 | 2.5 | 25.7 ^1^ | 3.3 |
| 12 | silla | chair | 35 | 29 | 0 | 0 | 8.6 ^1^ | 3 |
| 13 | paño | cloth | 35 | 11 | 0 | 0 | 0 | 0 |
| 14 | uña | nail | 35 | 11 | 0 | 0 | 11.4 | 3.9 |
| 15 | pelota | ball | 35 | 9 | 0 | 0 | 65.7 ^1^ | 3.3 |
| 16 | lápiz | pencil | 32 | 18 | 0 | 0 | 0 | 0 |
| 17 | comer | eat | 32 | 12 | 0 | 0 | 28.1 | 3.9 |
| 18 | gorra | cap | 32 | 15 | 0 | 0 | 6.3 ^1^ | 3.5 |
| 19 | mano | hand | 32 | 18 | 0 | 0 | 0 | 0 |
| 20 | virus | virus | 32 | 17 | 0 | 0 | 3.1 ^2^ | 2 |
| 21 | edificio | building | 32 | 15 | 9.4 | 3.7 | 18.8 | 4 |
| 22 | ropa | clothes | 32 | 17 | 3.1 | 4 | 15.6 | 4.4 |
| 23 | arena | sand | 32 | 10 | 0 | 0 | 37.5 ^2^ | 4.3 |
| 24 | lluvia | rain | 32 | 19 | 3.1 | 1 | 3.1 | 3 |
| 25 | barco | ship | 32 | 11 | 0 | 0 | 6.3 | 4.5 |
| 26 | alcohol | alcohol | 32 | 20 | 0 | 0 | 6.3 | 4.5 |
| 27 | caja | box | 32 | 21 | 0 | 0 | 21.9 ^2^ | 3.8 |
| 28 | hablar | talk | 32 | 19 | 0 | 0 | 3.1 | 2 |
| 29 | correr | run | 32 | 23 | 0 | 0 | 6.3 | 4.5 |
| 30 | llorar | cry | 32 | 8 | 0 | 0 | 87.5 ^1^ | 4.9 |
| 31 | serpiente | snake | 48 | 34 | 0 | 0 | 12.5 | 3.9 |
| 32 | ratón | mouse | 48 | 35 | 0 | 0 | 0 | 0 |
| 33 | perfume | perfume | 48 | 14 | 4.2 | 3 | 66.7 | 4.2 |
| 34 | león | lion | 48 | 17 | 2.1 | 5 | 0 | 0 |
| 35 | noche | night | 48 | 17 | 0 | 0 | 4.2 ^2^ | 3.3 |
| 36 | oro | gold | 48 | 23 | 2.1 | 5 | 20.8 ^2^ | 3 |
| 37 | zapato | shoe | 48 | 30 | 0 | 0 | 31.3 ^2^ | 3.5 |
| 38 | maleta | suitcase | 48 | 6 | 0 | 0 | 25 | 4.6 |
| 39 | hoja | sheet | 48 | 16 | 0 | 0 | 0 | 0 |
| 40 | bate | bat | 48 | 29 | 0 | 0 | 2.1 ^2^ | 5 |
| 41 | perro | dog | 48 | 10 | 0 | 0 | 27.1 | 4.5 |
| 42 | bar | bar | 48 | 23 | 4.2 | 5 | 29.2 ^1^ | 3.7 |
| 43 | casa | house | 48 | 28 | 0 | 0 | 4.2 ^2^ | 2.5 |
| 44 | cesta | basket | 48 | 18 | 0 | 0 | 10.4 | 3.9 |
| 45 | comida | food | 48 | 9 | 0 | 0 | 83.3 ^1^ | 4.6 |
| 46 | recuerdos | memories | 40 | 18 | 0 | 0 | 55 | 3.5 |
| 47 | llave | key | 40 | 22 | 0 | 0 | 35 | 4.5 |
| 48 | gato | cat | 40 | 18 | 0 | 0 | 0 | 0 |
| 49 | regalo | gift | 40 | 9 | 0 | 0 | 75 | 4.8 |
| 50 | botella | bottle | 40 | 17 | 0 | 0 | 40 | 4.7 |
| 51 | cuadros | paintings | 40 | 24 | 0 | 0 | 17.5 ^1^ | 4.9 |
| 52 | dolor | pain | 40 | 14 | 0 | 0 | 37.5 ^1^ | 4.1 |
| 53 | cansancio | tiredness | 40 | 19 | 0 | 0 | 27.5 | 4.1 |
| 54 | tráfico | traffic | 40 | 26 | 0 | 0 | 10 | 4.2 |
| 55 | pez | fish | 40 | 8 | 0 | 0 | 82.5 ^1^ | 4.6 |
| 56 | armas | weapons | 40 | 23 | 0 | 0 | 32.5 ^2^ | 4.6 |
| 57 | libros | books | 40 | 22 | 0 | 0 | 10 ^2^ | 4.8 |
| 58 | refresco | refreshment | 40 | 15 | 0 | 0 | 55 | 4.8 |
| 59 | falda | skirt | 40 | 8 | 0 | 0 | 67.5 ^2^ | 4.7 |
| 60 | avión | plane | 40 | 6 | 0 | 0 | 67.5 | 4.7 |
| 61 | coche | car | 33 | 15 | 0 | 0 | 33.3 | 4 |
| 62 | lechuga | lettuce | 33 | 16 | 0 | 0 | 24.2 | 4.1 |
| 63 | estantería | shelf | 33 | 10 | 0 | 0 | 45.5 | 4.2 |
| 64 | tarta | cake | 33 | 17 | 0 | 0 | 3 | 4 |
| 65 | pañuelo | handkerchief | 33 | 17 | 0 | 0 | 9.1 | 3 |
| 66 | antipático | unfriendly | 33 | 17 | 0 | 0 | 39.4 | 3.9 |
| 67 | chocolate | chocolate | 33 | 17 | 0 | 0 | 9.1 ^2^ | 4.3 |
| 68 | guante | glove | 33 | 6 | 0 | 0 | 24.2 | 4.3 |
| 69 | muro | wall | 33 | 18 | 0 | 0 | 33.3 | 3.8 |
| 70 | rueda | wheel | 33 | 21 | 9.1 | 3.7 | 18.2 ^1^ | 3.8 |

^1^ Summed percentages of words that are included in the category label, such as "*door*" and "*close*" for "*Things that can be used to prevent a door from closing*”.

^2^ Summed percentages of words not included in the category label but that express part of its essence, such as “*illegal*”, “*mafia*” and “*trafficking*” for “*Things that can be bought on the black market in Russia*”.
